# Global transcriptomic changes in glomerular endothelial cells in mice with podocyte depletion and glomerulosclerosis

**DOI:** 10.1038/s41419-021-03951-x

**Published:** 2021-07-09

**Authors:** Jia Fu, Zhengzi Yi, Minchao Cai, Weijie Yuan, Weijia Zhang, Kyung Lee, John Cijiang He

**Affiliations:** 1 Division of Nephrology, Department of Medicine, Icahn School of Medicine at Mount Sinai, New York, NY USA; 2 Department of Nephrology, Shanghai General Hospital, Shanghai Jiao Tong University, Shanghai, China; 3 Renal Program, James J. Peters Veterans Affairs Medical Center at Bronx, New York, NY USA

**Keywords:** Glomerular diseases, Experimental models of disease

## Abstract

Podocytes are a key component of the glomerular filtration barrier, and its dysfunction and eventual loss drive glomerular disease progression. Recent research has demonstrated the importance of podocyte cross-talk with other glomerular cells, such as glomerular endothelial cells (GECs), in both glomerular homeostasis and in disease settings. However, how GECs are affected globally by podocyte injury and loss in disease settings remains unclear. Therefore, to characterize the molecular changes occurring in GECs in response to the podocyte loss, we performed the transcriptomic profiling of isolated GECs after diphtheria toxin (DT)-mediated podocyte depletion in transgenic mice with podocyte-specific human DT receptor and endothelial-specific enhanced yellow fluorescent protein (EYFP) expression. DT administration led to nearly 40% of podocyte loss with the development of glomerulosclerosis. Differential gene expression analysis of isolated GECs in the diseased mice showed significant changes in pathways related to cell adhesion and actin cytoskeleton, proliferation, and angiogenesis, as well as apoptosis and cell death. However, quantification of EYFP + GECs indicated that there was a reduction in GECs in the diseased mice, suggesting that despite the ongoing proliferation, the concomitant injury and the activation of cell death program results in their overall net loss. The upstream regulator analysis strongly indicated the involvement of p53, TGF-β1, and TNF-α as key mediators of the molecular changes occurring in GECs in the diseased mice. Our findings demonstrate significant molecular changes in GECs as a secondary consequence of podocyte loss and provide a valuable resource for further in-depth analysis of potential glomerular cross-talk mediators.

## Introduction

Podocyte dysfunction and loss are major causes of proteinuria and are associated with the the progression of many glomerular diseases [1, 2]. Approximations from experimental models of glomerulosclerosis have indicated that about 20% of podocyte depletion is sufficient to cause glomerulosclerosis with mild, but sustained proteinuria, and that further depletion to 40% results in intense proteinuria and renal dysfunction [3, 4]. Recent studies also indicate that the cross-talk between glomerular cells is critical to maintain glomerular homeostasis, and that the alteration in such cross-talk might be a key process leading to the progression of glomerular disease [5–8]. In this regard, several angiogenic factors are implicated in the cross-talk between podocytes and glomerular endothelial cells (GECs). For example, podocyte-secreted vascular endothelial growth factor-A (VEGF-A) is required for maintaining the normal GEC function [9] and the alteration of its signaling pathway leads to thrombotic microangiopathy and is implicated in diabetic nephropathy pathogenesis [10, 11]. Podocyte-secreted endothelin-1 was also shown to be an important cross-talk mediator that results in increased oxidative stress in GECs and worsening of glomerular disease progression [12]. However, a broader view of how podocyte loss affects GECs had not been determined. Therefore, in this study, we undertook an unbiased approach of transcriptomic analysis of isolated GECs in mice expressing nuclear enhanced yellow fluorescent protein (EYFP) transgene expression under the control of *Flk-1* element for effective isolation of GECs [13–15] and podocyte-specific human diphtheria toxin (DT) receptor transgenes for effective dose-dependent podocyte depletion upon DT administration as a well-established model of focal segmental glomerulosclerosis (FSGS) [4, 16]. Using this mouse model, we now provide the transcriptomic analysis of molecular changes occurring in GECs in response to podocyte dysfunction and loss in experimental FSGS.

## Materials and methods

### Transgenic mice and in vivo podocyte depletion

All mouse studies were performed under the guidelines of and approved by the Institutional Animal Care and Use Committee at the Icahn School of Medicine at Mount Sinai (New York, NY). Mice were housed in a specific pathogen-free facility with free access to chow and water, and a 12 h day/night cycle. The Flk1::H2B-EYFP transgenic reporter mice (Flk1-EYFP) that express nuclear EYFP under the control of *Flk-1* promoter and intronic enhancer in the C57BL/6J background was previously described [13–15]. The iDTR (C57BL/6-Gt(ROSA)26Sortm1(HBEGF)Awai/J) mice were purchased from The Jackson Laboratory (Bar Harbor, ME). To selectively induce DTR expression in podocytes, iDTR mice were crossed with *Nphs2-Cre* mice (a generous gift from Dr. Lawrence Holzman, University of Pennsylvania) to generate iDTR;*Nphs2*-Cre mice and further crossed with Flk1-EYFP mice to generate iDTR;*Nphs2*-Cre;*Flk1*-EYFP (Pod-DTR;Flk1-EYFP) mice. For podocyte depletion, DT (Sigma-Aldrich) was dissolved in sterile phosphate-buffered saline and was administered intraperitoneally in Pod-DTR;Flk1-EYFP mice at a concentration of 5 ng/g body weight to achieve ~40–45% loss of podocyte depletion in 10 days [16]. Both male and female double transgenic mice aged 8 weeks were used.

### Measurement of urinary albumin-to-creatinine ratio

Urine creatinine was quantified by using commercial kits from BioAssay Systems (Hayward, California, USA). Urine albumin was determined by using a commercial assay from Bethyl Laboratory, Inc. (Houston, Texas, USA). Urine albumin excretion was expressed as the ratio of urine albumin to creatinine [17].

### Kidney histology

Harvested kidney samples for histology were fixed in 10% formalin, embedded in paraffin, and cut into 4 µm sections. Periodic Acid–Schiff-stained sections were used for the assessment of kidney histology. The number for H2B-EYFP-positive cell nuclei was quantified representing endothelial cells. Quantification of EYFP + GECs per glomerular cross-section was based on a minimum of 50 glomeruli per experimental group (*n* = 3 mice per group) in a blinded manner, under ×400 magnification (Zeiss Axioimager IE microscope).

### mRNA isolation from EYFP + GECs

Glomeruli were first isolated by perfusion of magnetic beads (Dynabeads) and EYFP-labeled endothelial cells were sorted from isolated glomeruli using the same protocol as previously described [18]. On average, 200,000 GECs were obtained per mouse. Isolated GECs were pooled (GECs from four mice per sample, three samples each per experimental group) and total mRNA from each sample was isolated by using RNeasy mini kit (Qiagen 74104) with on-column DNase digestion according to the manufacturer’s protocol. RNA concentrations were quantified using a Nanodrop Spectrophotometer at a wavelength of 260 nm. RNA samples were then analyzed by Bioanalyzer at a concentration of 100–200 ng/μl to verify the concentration and the purity of samples. Only the samples with RNA integrity values > 7.5 were used for mRNA sequencing at the CLC Genomics and Epigenomics Core Facility at Weil Cornell Medical College.

### Bioinformatics analysis of RNA-seq data

The RNA sequencing (RNA-seq) data were analyzed by the following procedure. Briefly, after sequence quality filtering at a cutoff of a minimum quality score Q20 in at least 90% bases, the good quality reads were aligned to the University of California Santa Cruz *Mus musculus* reference genome and transcriptome (build mm10) using the Burrows-Wheeler Aligner (bwa) [19]. The reads that are uniquely aligned to the exon and splicing-junction sites for each transcript were combined to calculate as expression level for a corresponding transcript and further normalized based on reads per kilobase per million reads to compare transcription levels among samples [20]. Gene expression value was transformed into the log2 base scale. Principal component analysis (PCA) was first performed to assess the sample correlations using the expression data of all the genes. The differentially expressed genes (DEGs) in antibody-injected mice compared to vehicle control mice were identified by the R package DEGseq [21] and limma test [22] for endothelial transcriptome studies. A specific gene was deemed to be significantly differentially expressed if the *p*-value given by these methods was ≤0.05. Then we did Gene Ontology (GO) and pathway analysis for the DEGs using INGENUITY^©^ IPA (http://www.ingenuity.com/products/ipa) and online tool Enrichr [23]. The read coverage of gene functional elements was also visualized by the Integrative Genome Viewer tool (http://www.broadinstitute.org/igv/) from the genome alignment file. Heatmap analysis was performed for the top fold-changed 50 differential expression genes after the median center transformed with Multi-Experiment Viewer software [24].

### Immunofluorescence staining

Staining, image acquisition, and fluorescence intensity quantification were performed as previously described. Frozen sections were incubated with Unconjugated AffiniPure Fab Fragment Goat Anti-Mouse IgG (H + L) (Jackson ImmunoResearch Labs, 115-007-003) for 1 h at room temperature after normal serum blocking, to eliminate background staining. Then, immunofluorescence staining was performed using the following antibodies: rabbit anti-WT1 (sc-192, Santa Cruz Biotechnology), mouse anti-SPP1 (secreted phosphoprotein), (MPIIIB10, Developmental Studies Hybridoma Bank), mouse anti-CARP/ANKRD1 (ankyrin repeat domain 1) (sc365056, Santa Cruz Biotechnology), and rabbit polyclonal Cleaved Caspase 3 (9664, Cell Signaling). Isolectin GS-IB4 (121413, Invitrogen) was also used to detect endothelial cells. Slides were counterstained with 4′,6-diamidino-2-phenylindole and mounted with the use of Vectashield hardset antifade mounting medium (Vectorlabs). Fluorescence images were acquired using the Axioplan 2 IE microscope (Zeiss).

### Gene silencing in GECs

For knockdown of *Spp1* and *Ankrd*1, five independent pLKO.1 lentiviral short harpin RNA (shRNA) plasmids for m*Spp1* and m*Ankrd1* were purchased from Sigma-Aldrich; pLKO.1 lentivector encoding scrambled shRNA was used as a negative control. Together with psPAX2 packaging and Vesicular stomatitis virus glycoprotein (VSV-G) envelope plasmids, pLKO.1 plasmids were transfected in the human embryonic kidney-293T cells with the use of PolyJet transfection reagent (SignaGen Laboratories, Rockville, MD) according to the manufacturer’s manual. Supernatant was collected 48 h post transfection, harvested, and concentrated. mouse glomerular endothelial cells (mGECs) grown at 33 °C were transduced with corresponding lentivirus in the presence of 8 μg/ml Polybrene (Sigma-Aldrich) at a multiplicity of infection of 5 and selected for puromycin resistance. Stably transduced mGECs were cultured at 37 °C for ≥7 days prior to experiments.

### Western blotting

Cells were homogenized in NP-40 lysis buffer containing protease and phosphatase inhibitor cocktail. Equal amounts of protein samples were separated on SDS polyacrylamide gel, transferred to polyvinylidene fluoride membranes (Millipore), and probed with primary antibodies: mouse anti-SPP1 (MPIIIB10, Developmental Studies Hybridoma Bank), mouse anti-CARP/ANKRD1 (sc365056, Santa Cruz Biotechnology), rabbit polyclonal Cleaved Caspase 3 (9664, Cell Signaling), and rabbit anti-GAPDH (2118; Cell Signaling Technologies).

### In vitro Matrigel GEC tube formation assay

Early passaged mGECs were differentiated at 37 °C for 5 days and induced with 30 mM high glucose or high mannitol (25 mM mannitol + 5 mM glucose) in EGM2 medium (Promo Cell) containing 1% fetal bovine serum for an additional 72 h. mGECs were then trypsinized and replated on top of a Matrigel (Corning, Corning, NY) bed in a 96-well plate (1.5 × 10^5^ cells/ml) as described previously [14, 25]. After 6–8 h of plating, the endothelial tube formation was visualized using the inverted Leica DMI8 microscope (Leica Microsystems, Buffalo Grove, IL).

### Statistical analysis

Data are expressed as mean ± SD. For comparison of means between three or more groups, two-way analysis of variance with Bonferroni post test was applied. For comparisons of means between two groups, two-tailed unpaired *t*-tests with Welch’s correction were performed. Prism 8 (GraphPad, La Jolla, California, USA) was used for statistical analyses.

## Results

### DT-mediated podocyte depletion and glomerulosclerosis in *Flk1*-EYFP transgenic mice

To facilitate the isolation of GECs, we utilized the Flk1-EYFP transgenic mice that express the nuclear-localized EYFP under the regulatory elements of *Flk1* gene [13–15] that was crossed with transgenic mice with podocyte-specific human DT receptor expression (Pod-DTR) to generate the Pod-DTR;Flk1-EYFP mice. Podocyte depletion was achieved by administration of DT as previously described [16, 26]. DT-injected age-matched Flk1-EYFP mice without DTR expression were used as controls. All mice were killed at 10 days post-DT injection for analysis.

**Fig. 1:**
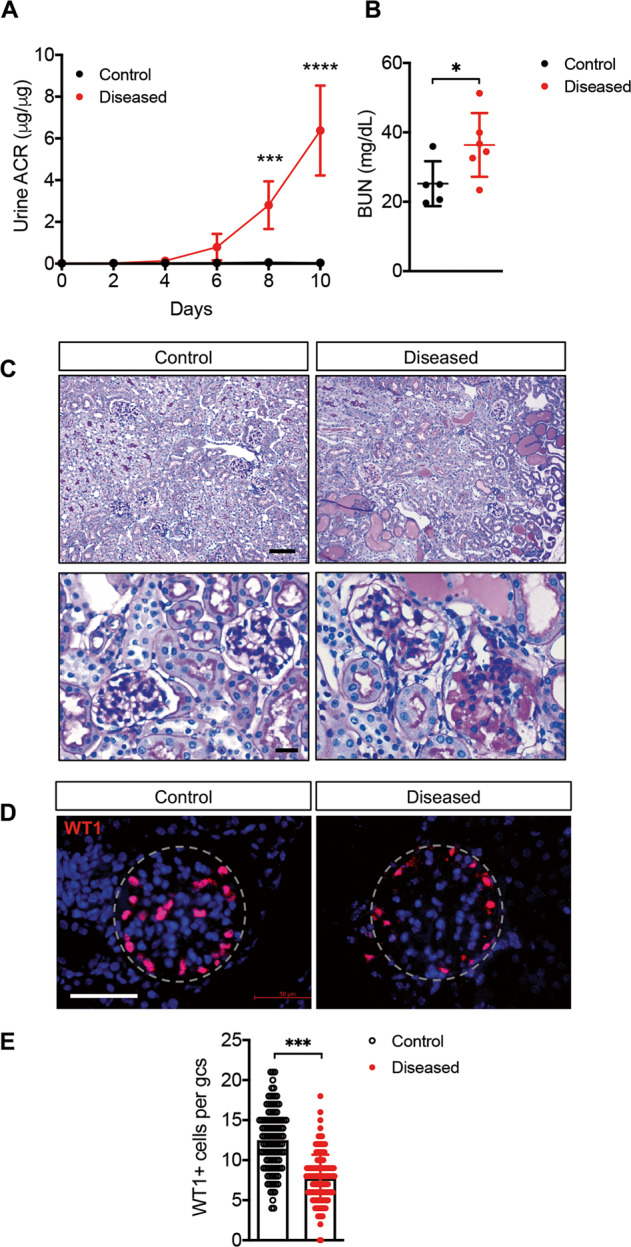
DT-induced podocyte depletion in Flk1-EYFP mice. **A** Urinary albumin-to-creatinine (UACR) ratio in samples collected after DT injection (*n* = 5–6 mice per group). ***p < 0.001 and ****p < 0.0001 when compared between groups by two-way ANOVA with Bonferroni’s correction. **B** Blood urea nitrogen (BUN) levels at 10 days after DT injection (*n* = 5 control, *n* = 6 diseased). *P < 0.05 between groups by unpaired two-tailed *t*-test with Welch’s correction. **C** Representative images of periodic acid–Schiff (PAS)-stained kidneys in low (top panels) and high (bottom panels) magnifications. Scale bars, 100 μm (top) and 20 μm (bottom). **D** Representative images of WT1 immunostaining of kidney sections. Kidney sections were counterstained with Hoechst (*n* = 3 mice per group; glomeruli are outlined with dotted lines). Scale bar, 50 μm. **E** Quantification of WT1-positive cell per glomerular cross-section (gcs) (*n* = 3 mice per group with at least 60 glomeruli counted per mouse). ***p < 0.001 between groups by unpaired two-tailed *t*-test with Welch’s correction.

As anticipated, DT administration in Pod-DTR;Flk1-EYFP mice (referred to as “diseased”) resulted in a progressive increase in the urinary albumin-to-creatinine ratio over time and elevated blood urea nitrogen levels at 10 weeks post injection in comparison to DT-injected Flk1-EYFP mice (referred to as “control”) (Fig. 1A, B). However, DT injection did not significantly alter the body weight of mice in either group (Supplementary Fig. 1). Kidneys of diseased mice displayed histopathological features typical of FSGS (Fig. 1C). As anticipated, the quantification of podocytes per glomerular cross-section using WT1 immunofluorescence indicated that DT injection resulted in ~40% of podocyte loss (12.5 ± 3.8 in control vs. 7.7 ± 2.9 in diseased, *p* < 0.001, Fig. 1D, E).

### Transcriptomic analysis suggests angiogenic and cell death response in GECs in diseased mice

**Fig. 2:**
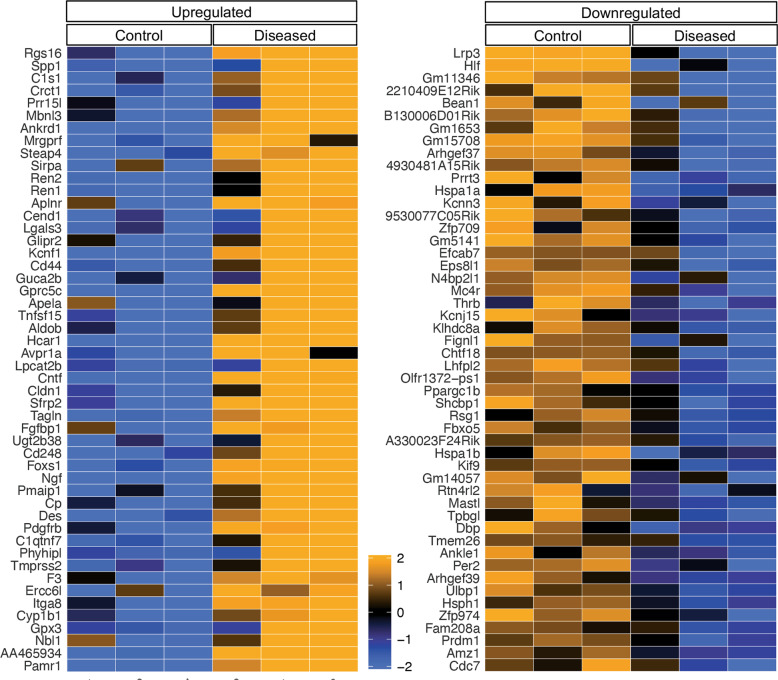
Differentially expressed genes in isolated GECs from diseased vs. control mice. Heatmap of top 50 genes that are upregulated (left) or downregulated (right) in the diseased vs. control GECs. Each column represents genes from a control or diseased mouse sample (*n* = 3 samples per group, where each sample represent pooled GECs from four mice).

For the transcriptomic analysis of GECs, isolated glomeruli from experimental mice were dissociated and EYFP^+^ cells were sorted as described previously [14]. GECs obtained from four mice were pooled and processed as a single sample for bulk RNA-seq and three samples were analyzed per experimental group, such that each experimental group consisted of sorted GECs from 12 mice. The principal component analysis of RNA-seq data showed distinct clustering of samples between diseased vs. control mice (Supplementary Fig. 2). Figure 2 shows the heatmap of top 50 DEGs that are upregulated or downregulated in the GECs from the diseased mice vs. controls (full list of DEGs is provided in Supplementary Excel File 1). Interestingly, the top upregulated genes included *Rgs16*, *Spp1*, and *Ankrd1* (Fig. 2), which are implicated in endothelial activation and angiogenic response. RGS16 (regulator of G-protein signaling 16) is a modulator of Gα13 signaling [27], a signaling cascade implicated in the induction of angiogenesis [28–30]. SPP1 (also known as osteopontin), which has been described to regulate bone metabolism in osteoclasts and chemotactic responses in various immune cells [31, 32], was also shown to induce angiogenesis in endothelial cells [33, 34]. Notably, the plasma level of osteopontin is associated with diabetic nephropathy progression in both type 1 and type 2 diabetes [35, 36], and the genetic ablation of osteopontin attenuated diabetic glomerulopathy in the mouse models of type 1 and type 2 diabetes [37]. Importantly, *SPP1* was also found be upregulated in micro-dissected glomeruli of human FSGS patients when compared with normal control kidney samples [38] (Supplementary Fig. 4). In addition to these three genes, many significantly upregulated DEGs in the diseased mice included secreted ligands that modulate endothelial activation and/or homeostasis, such as *Cntf* (ciliary neurotrophic factor), *Ngf* (nerve growth factor), *Ctgf* (connective tissue growth factor), *Tgfb3* (transforming growth factor-β3), *Vegfd* (vascular endothelial growth factor d), *Edn1* (endothelin-1), and *Lrg1* (leucine-rich α-2 glycoprotein 1) (Supplementary Excel File 1). We previously showed that LRG1 potentiates diabetes-induced angiogenesis in GECs during early diabetic kidney disease [15].

Among the top downregulated genes were *Lrp3* (low-density lipoprotein receptor-related protein 3), whose role is implicated in the differentiation of bone marrow stromal cells but in endothelial cells is not clear, and *Arhgef37* and *Arhgef39* (Rho guanine nucleotide exchange factor 37 and 39) that are involved in the clathrin-mediated endocytic process [39] and cell migration [40], respectively (Fig. 2 and Supplementary Excel File 1).

**Fig. 3:**
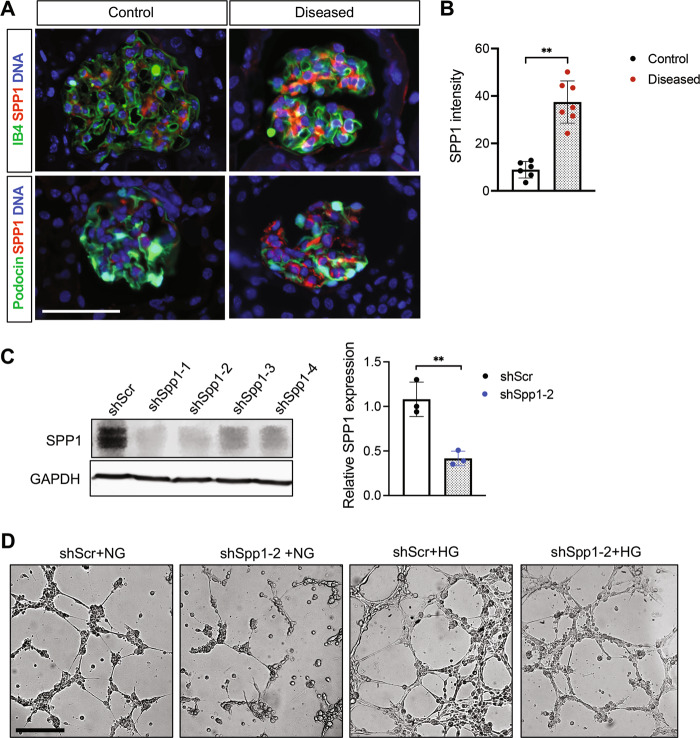
SPP1 is increased in GECs in FSGS and contributes to angiogenic response. **A** Representative immunofluorescence images of SPP1 (red) with endothelial marker isolectin GS-IB4 (IB4, green, top panels) or podocyte marker, podocin (green, bottom panels). DNA is counterstained in blue. Scale bar, 50 μm. **B** Average fluorescence intensity of SPP1 in glomeruli of control vs. diseased mice are shown in arbitrary units (*n* = 6 control, 7 diseased mice, 20–30 glomeruli quantified per mouse). **C** Western blot analysis of cultured GECs expressing short harpin RNA (shRNA) against scrambled control (shScr) or against four independent clones of shRNA against *Spp1* (ShSpp1-1, -2, -3, and -4). The densitometric analysis of SPP1 normalized to GAPDH is shown for shScr vs. shSpp1-2 (*n* = 3 independent experiments). **D** In vitro angiogenesis assay of GECs cultured in normal glucose (NG, 5 mM) or high glucose (HG, 25 mM) and plated on Matrigel bed. Images were taken 6 h post plating. Scale bar, 250 μM. **P < 0.01 between two groups by unpaired two-tailed *t*-test.

To confirm some of the above observations, we chose SPP1 and ANKRD1 for further validation. As shown in Fig. 3A, B, SPP1 expression was enhanced in the glomeruli of diseased mice that largely overlapped with isolectin GS-IB4-positive GECs rather than podocin-positive podocytes. To further confirm the angiogenic role of SPP1 in GECs, we cultured mGECs stably expressing shRNA against Spp1 or scrambled control sequence (Fig. 3C), and tested for its effects under high glucose-potentiated angiogenic response, as we have done previously [15]. Indeed, the reduction in *Spp1* expression attenuated the high glucose-induced angiogenesis in vitro (Fig. 3D).

**Fig. 4:**
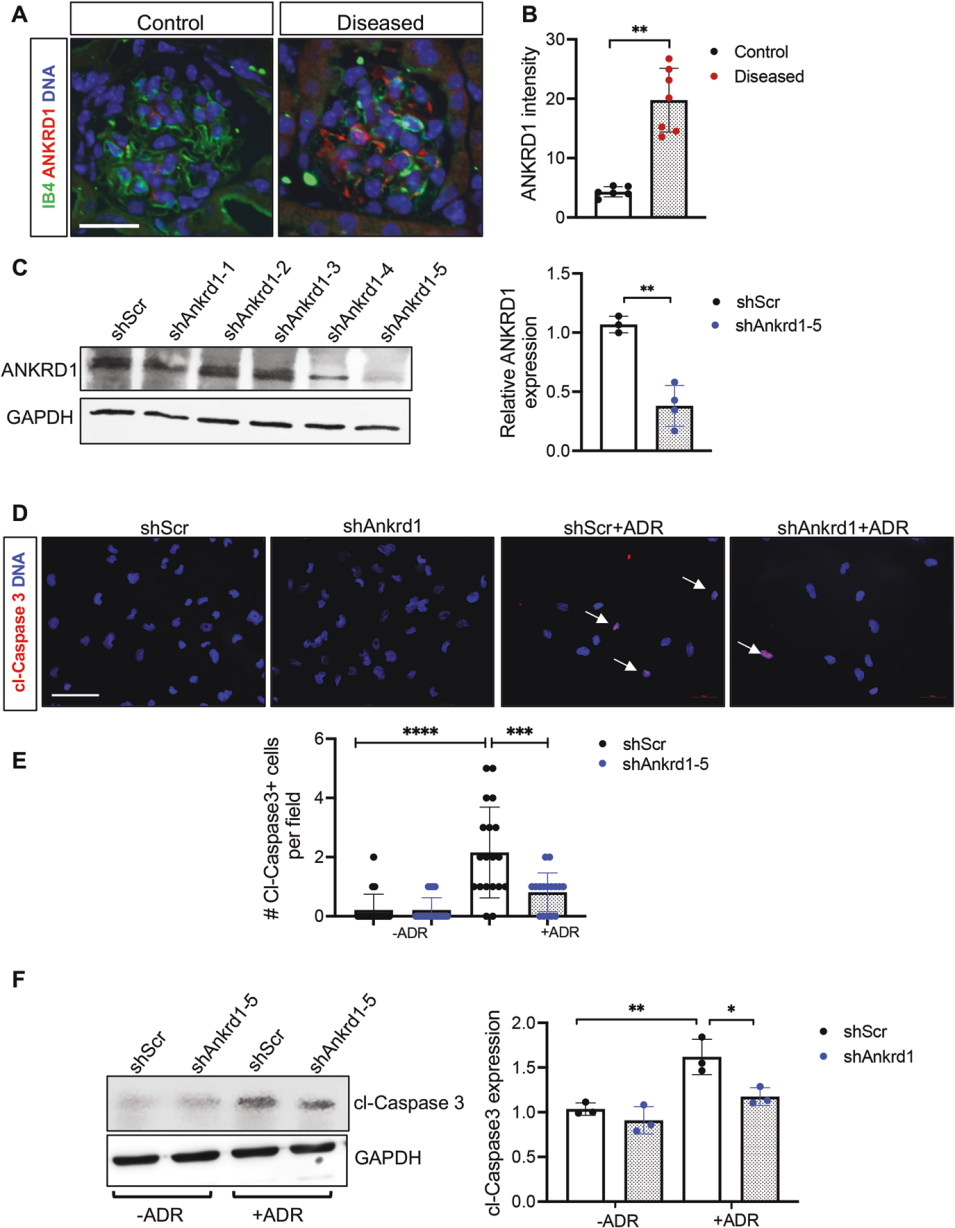
ANKRD1 is increased in GECs in FSGS and contributes to endothelial apoptosis. **A** Colocalization of ANKRD1 with endothelial marker isolectin GS-IB4 in control and podocyte-depleted diseased mice. Scale bars, 25 μm. **B** Quantification of glomerular ANKRD1 immunofluorescence intensity (in arbitrary units) in control and diseased mice (*n* = 6 control mice, *n* = 7 diseased mice, with at least 30 glomeruli evaluated per mouse). **P < 0.01 between two groups by unpaired two-tailed *t*-test. **C** Western blot analysis of cultured GECs expressing short harpin RNA (shRNA) against scrambled control (shScr) or against *Ankrd1* (Sh*Ankrd1*-1, -2, -3, -4, and -5, five independent clones) are shown on the left. The densitometric analysis of ANKRD1 normalized to GAPDH is shown for shScr vs. ShAnkrd1-5. **D** Representative images of cleaved Caspase 3 (cl-Caspase 3) staining in cultured mGECs with or without adriamycin treatment (500 ng/mL) for 24 h. Scale bar, 25 μm. Arrowhead show examples of cl-Caspase 3 positive cells. **E** Quantification of cl-Caspase 3 positive cells (*n* = 3 independent experiments, with 20 fields analyzed per group). **F** Western blot analysis of cl-Caspase 3 in mGECs with or without ADR treatment. Quantification of cl-Caspase 3 levels is shown on the right. *P < 0.05, **P < 0.01, ***P < 0.001, and ****P < 0.0001 between indicated groups by two-way ANOVA with Tukey’s multiple comparison test.

ANKRD1 is a profibrotic transcriptional cofactor that is sharply induced during wound repair and reported to induce angiogenesis in endothelial cells [41, 42]. Although ANKRD1 was initially reported to be upregulated in podocytes in various human kidney diseases, including crescentic glomerulonephritis, diabetic nephropathy, and lupus nephritis [43, 44], a recent report showed ANKRD1’s involvement in vascular injury [45]. Consistent with these observation, glomerular ANKRD1 was increased in the glomeruli of diseased mice (Fig. 4A, B), which overlapped with IB4-endothelial cells, as well as neighboring cells, likely to be remaining podocytes. As ANKRD1 was shown previously to participate as a co-activator of p53 in the induction of apoptosis [46], we examined the effects of ANKRD1 knockdown in Adriamycin-induced apoptosis in mGECs in vitro (Fig. 4C). As shown in Fig. 4D–F, knockdown of *Ankrd1* significantly inhibited the expression of cleaved Caspase 3, as a surrogate marker of apoptosis after treatment with Adriamycin. These results support the role of ANKRD1 in enhancing GEC apoptosis. Together, these results suggest that both endothelial activation and apoptosis may be occurring concomitantly in GECs in the setting of podocyte loss in FSGS.

### Podocyte loss results in GEC injury and loss

**Fig. 5:**
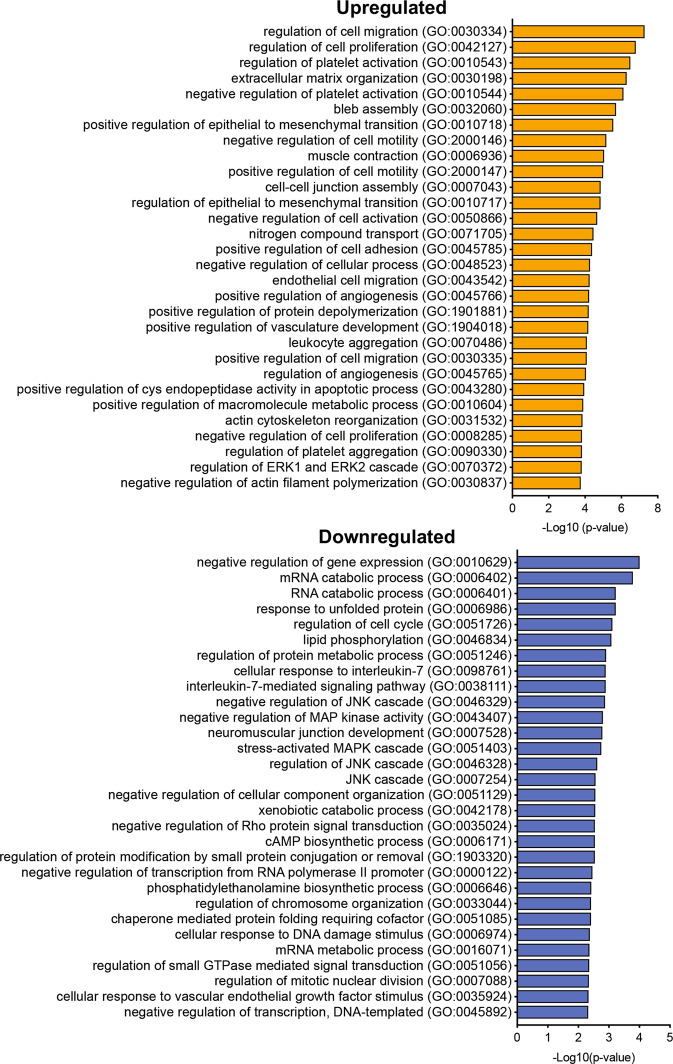
Gene Ontology (GO) pathway analysis of differentially genes in isolated GECs from diseased vs. control mice. Top 30 pathways of upregulated (top) or downregulated (bottom) DEGs are shown.

**Fig. 6:**
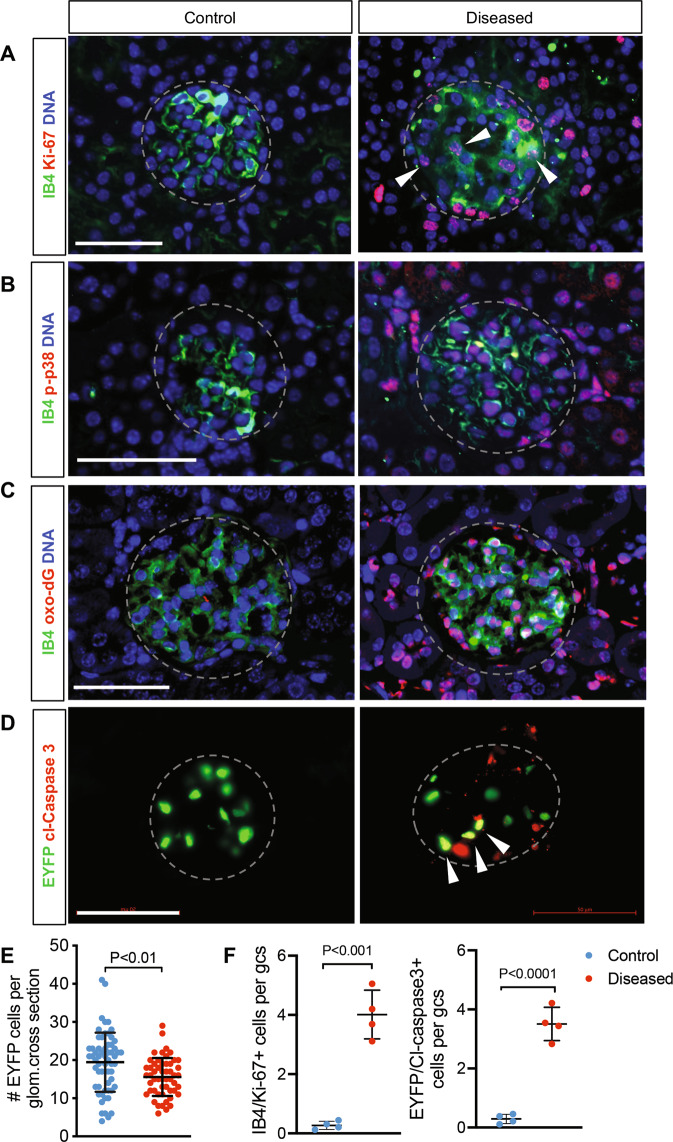
Podocyte loss results in net GEC loss. **A**–**D** Representative immunofluorescence images of control and diseased glomeruli as indicated. Glomeruli are outlined in dotted lines. Scale bars, 50 μm. DNA is counterstained in blue. **A** Ki-67 (red) and endothelial marker isolectin GS-IB4 (IB4, green). Arrowheads show examples of Ki-67+ and IB4+ cells. **B** Phospho-p38 MAPK (p-p38, red) and IB4 (green). **C** DNA damage marker 8-oxo-2′-deoxyguanosine (oxo-dG, red) and IB4 (green). **D** Cleaved Caspase 3 (cl-Caspase 3, red) and EYFP marking the GECs. Arrowheads show examples of EYFP nuclei that are cl-Caspase 3+. DNA counterstain is omitted for clarity of double-positive nuclei in yellow. **E** Average number of EYFP+ cells per glomerular cross-section (*n* = 61 glomeruli from control mice, 53 from diseased mice, quantified from three mice per experimental group). *P* < 0.01 between groups by unpaired two-tailed *t*-test with Welch’s correction. **F** Average number of IB4/Ki-67 double-positive cells (left) and EYFP/cl-Caspase 3 double-positive cells per glomerular cross-section (gcs) in mice (*n* = 4 mice per group, 20–30 glomeruli evaluated per mouse). *P* < 0.001 and *P* < 0.0001 by unpaired two-tailed *t*-test.

We next analyzed the pathway enrichments from DEGs of diseased mice by utilizing the combination of gene-list libraries of GO biological processes, Kyoto Encyclopedia of Genes and Genomes, and Wiki-Pathway. The upregulated DEGs were largely associated with cell-matrix associations, cytoskeleton regulation, cell proliferation, and cell death pathways (Fig. 5, Supplementary Fig. 3, and Supplementary Excel File 2). The downregulated genes were associated with the negative regulation of gene expression and of stress-activated mitogen-activated protein kinase (MAPK) (i.e., p38 MAPK and JNK) signaling and cell cycle pathways. These results suggested that the modulation of endothelial cell activation and proliferation (both positive and negative), as well as cell death, may be concomitantly induced in GECs following the podocyte loss. Immunofluorescence staining of cell cycle marker Ki-67 indeed showed an increased presence of proliferating cells in the glomeruli of diseased mice, which included those that colocalized with endothelial marker isolectin GS-IB4 (Fig. 6A, arrowheads). As the above pathway analysis also indicated an increased apoptotic process (from upregulated DEGs) and decreased negative regulation of stress-activated MAPK/JNK pathways (from downregulated DEGs) (Fig. 5), we additionally examined the expression levels of phosphorylated p38 MAPK and cleaved Caspase 3. We also examined the expression of DNA damage marker 8-oxo-2′-deoxyguanosine, as an accumulation of reactive oxygen species (ROS) in GECs subsequent to podocyte injury has been observed [12]. We indeed observed an increased expression of phospho-p38 MAPK, 8-oxo-2′-deoxyguanosine, and cleaved Caspase 3, which largely colocalized with either isolectin GS-IB4 or EYFP (Fig. 6B–D, F). Therefore, to assess the net change in GECs, we quantified the number of EYFP + GECs in control and diseased mice. As shown in Fig. 6E, there was a net reduction in the average number of GECs per glomerular cross-section in the diseased mice vs. controls (19.44 ± 7.76 vs. 15.57 ± 4.99, *p* < 0.01). These results collectively suggest that although endothelial activation and proliferation are induced in the GECs in the setting of podocyte loss and FSGS, the concomitant injury and activation of cell death program results in their overall net loss and thereby contributing to further glomerular dysfunction.

### Activation of p53, TGF-β1, and TNF-α pathways are likely responsible for the molecular changes in the GECs of diseased mice

**Fig. 7:**
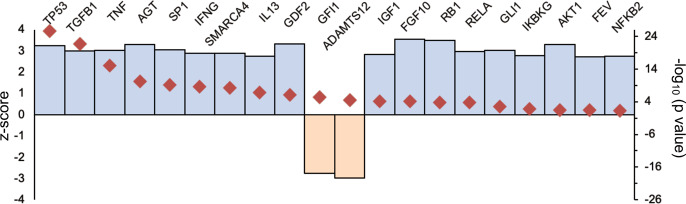
Activation *z-*scores and *P*-values of the top predicted upstream regulators from pathway analysis of DEGs. The blue column shows the *z*-score and red square shows the −log10 of overlap *p*-value for individual upstream activator.

We took advantage of the availability of causal analytics tool to predict the upstream regulator of the DEG expression, as described by Kramer et al. [47], by *z*-scores that infer the activation state of the putative regulator (activated or inhibited) and overlap *p*-values that measures the enrichment of regulated genes within the dataset. As shown in Fig. 7, the putative activators with the highest *z*-scores and overlap *p*-values were p53, transforming growth factor-β (TGF-β), and tumor necrosis factor-α (TNF-α). The only significant inhibitors were related to transcriptional regulation by GFI1 (growth factor independent 1 transcriptional repressor) and ADAMTS12 (a disintegrin and metalloproteinase with thrombospondin motifs 12) signaling. These results are consistent with the above observation of increased endothelial activation and cell death.

Taken together, our results indicate that podocyte loss results in subsequent GEC angiogenic response, as well as injury and loss, mediated likely through the activation of p53-, TGF-β-, and TNF-α-mediated pathways.

## Discussion

Recent studies suggest that the paracrine communication between glomerular cells is pivotal for the proper function of glomerular filtration barrier and their dysregulation may underlie pathologic events contributing to the progression of glomerular disease. Notable examples of podocyte to GEC cross-talk mediators are VEGF-A, which is abundantly expressed by podocytes that affect VEGFR-2 signaling in GECs [9, 48], and podocyte-derived angiopoietin system that signals through endothelial Tie-2 receptor to promote glomerular microvascular growth [49]. Thus, ensuing GEC injury is implicated in disease contexts where podocyte injury and loss occurs. However, a global change occurring in GECs as a consequence of podocyte loss had not been explored. To observe a secondary change in GECs as a consequence of podocyte loss as a primary insult, in this study we utilized an inducible model of DT-mediated podocyte depletion. We also leveraged the utility of Flk1-EYFP mice, which shows a prominent nuclear EYFP in GECs as compared to tubular endothelial cells [50] and thus amenable for efficient isolation of viable GECs for transcriptomic profiling analysis [14]. Therefore, we were able to determine how podocyte-specific injury and loss affected the global transcriptomic profile in GECs in vivo. To our knowledge, this is the first study to profile mRNA expression in GEC in the mouse model with podocyte depletion.

Analysis of the RNA-seq data revealed that DEGs were mostly involved in endothelial proliferation and angiogenesis, cytoskeletal reorganization and adhesion, and cell death. We have previously noted podocyte loss and accompanying TGF-β-mediated endothelial activation and proliferation in experimental models of diabetic nephropathy [15, 51], which may also occur in this model of FSGS. Interestingly, the downregulated pathways included the negative regulation of stress-activated MAPK signaling cascade, consistent with the increased cell death program. As for changes in cytoskeletal reorganization and adhesion, these may be due to significant shear stress in the diseased condition. Whether the GECs may undergo detachment leading to their loss, akin to podocyte detachment and loss in disease setting, remains to be determined. The quantification of GECs in the diseased mice experimentally confirmed the reduction of GECs in the diseased mice, despite endothelial activation and proliferation. This is further supported by the validation of two genes that are highly upregulated in GECs of diseased mice, namely SPP1, which enhanced angiogenesis, and ANKRD1, which augmented GEC apoptosis in cultured GECs. Although the mechanisms of GEC loss in vivo likely involve multiple signaling pathways, the upstream regulator analysis strongly indicates the involvement of p53, TGF-β1, and TNF-α signaling cascade. It is also plausible that cell death response is induced by accumulation of ROS and ensuing DNA damage response in GECs in glomerular disease, as previously demonstrated by us and others [14, 15, 51–53].

The findings from current works confirm significant molecular changes that occur in GECs as a secondary consequence of podocyte loss and offers the global transcriptomic profile of GECs in kidney disease in vivo. Moreover, we suspect that injured endothelial cells can further damage podocytes via paracrine mediators, thereby forming a vicious cycle to aggravate the progression of kidney disease. Therefore, the identification of these key mediators between these two cell types would be essential in developing better therapeutic targets to slow the progression of the glomerular disease. As we have identified DEGs and potential pathways involved based on these gene expression changes as a response to podocyte loss, future in-depth studies are required to delineate the role of specific genes and pathways involved in GEC injury in kidney disease.

Nevertheless, our current study has several limitations. First, we observed the global changes in GECs after DT-mediated podocyte depletion has occurred, rather than at earlier stages as podocytes are undergoing injury and loss. Therefore, there may be some secondary effects that could affect mRNA profiles of GECs such as changes of hemodynamic glomerular pressure or alteration of GBM components in addition to paracrine factors released by podocytes. The current analysis does not allow such distinction as causative factors for changes in endothelial cells. It would be informative in future studies to compare the gene expression profiles of GECs at different time points. Second, we did not study the role of individual secreted factors from podocytes in mediating this podocyte to GEC cross-talk. Third, although each sample contained pooled GECs sorted from four mice, the number of the sample size used for sequencing per group was relatively small, making it more difficult to control for experimental variation that may contribute to altered gene expression between groups. Regardless of these minor limitations, our study provides a unique resource for the research community for further in-depth analysis of potential glomerular cross-talk mediators in kidney disease that can help develop new therapeutic approaches for patients with glomerular disease.

## Supplementary information

Supplemental Figures

EXcel file 1

Excel File 2

## Data Availability

RNA-seq data are deposited in the Gene Expression Omnibus, GSE128736.
