# Supplementary material for: Global transcriptomic changes in glomerular endothelial cells in mice with podocyte depletion and glomerulosclerosis
Source: Cell Death Dis. 2021 Jul 9;12(7):687. doi: 10.1038/s41419-021-03951-x (PMC8270962; doi:10.1038/s41419-021-03951-x)
Supplement: Supplementary file 1 — Supplemental Figures [file 41419_2021_3951_MOESM1_ESM.pdf]

Supp. Figures

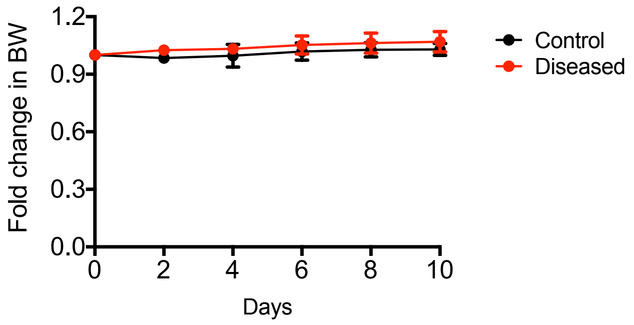

S. Fig. 1: Fold change in body weight of DT-injected mice. Body weight of mice are shown as a fold change from before and after DT injection (n=5-6 mice per group).

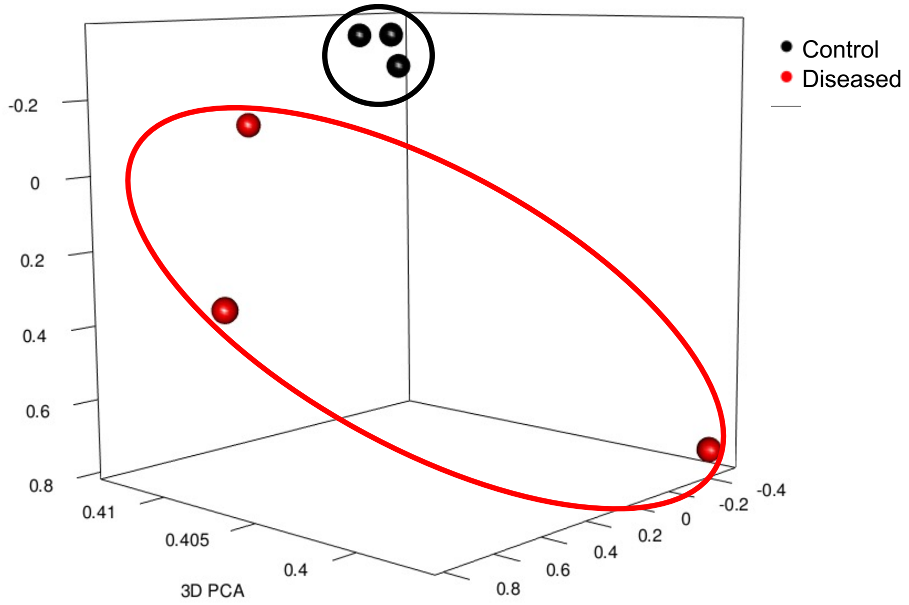

S. Fig. 2: Principal component analysis of genes from isolated GECs from control and diseased mice. Each sample represents genes from pooled GECs from 4 mice (n=3 samples per group, GECs pooled from 4 mice per sample).

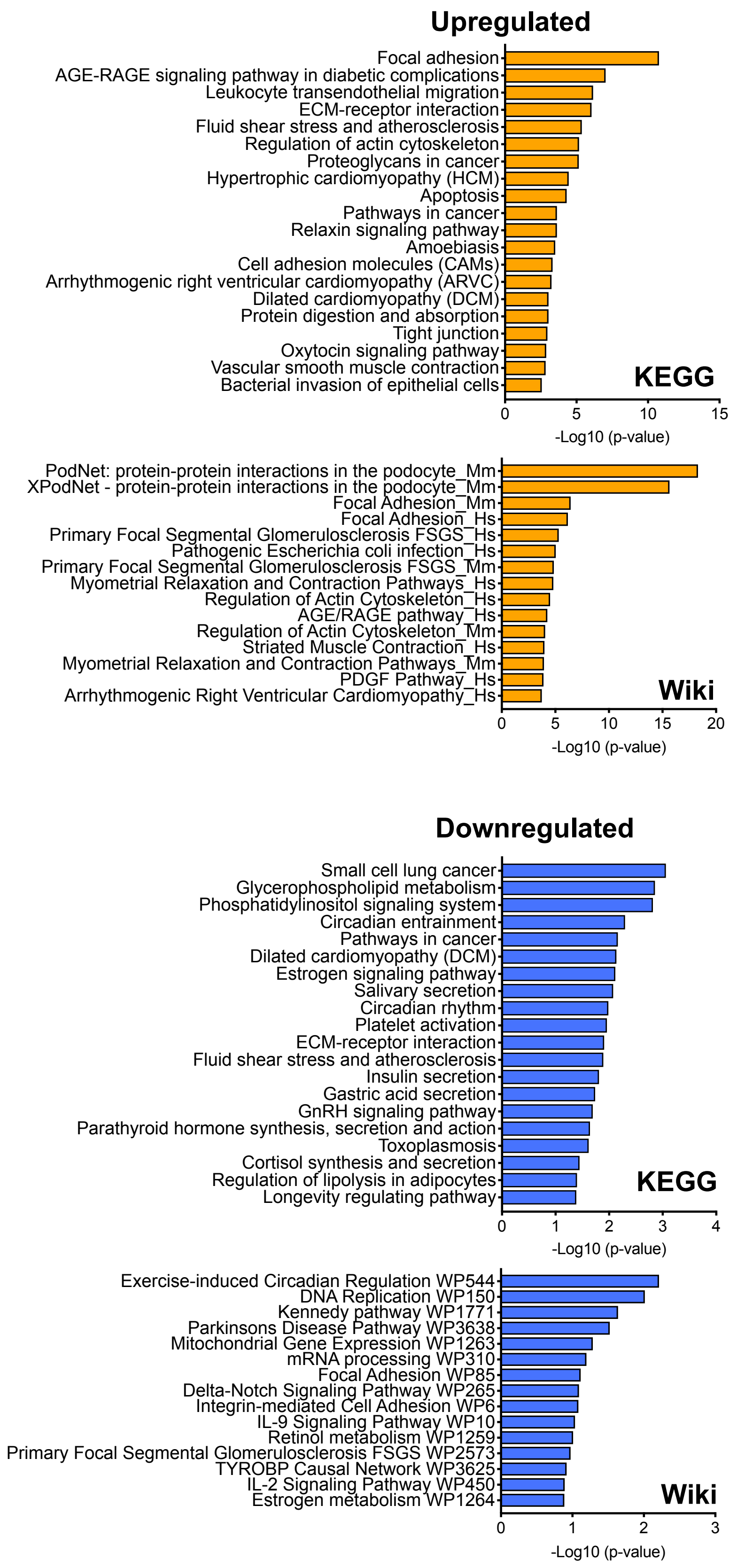

S. Fig. 3: Pathway analysis of differentially genes in isolated GECs from diseased vs control mice. Top 20 by KEGG pathway and top 15 Wiki pathway analyses of upregulated (top) and downregulated (bottom) DEGs are shown.

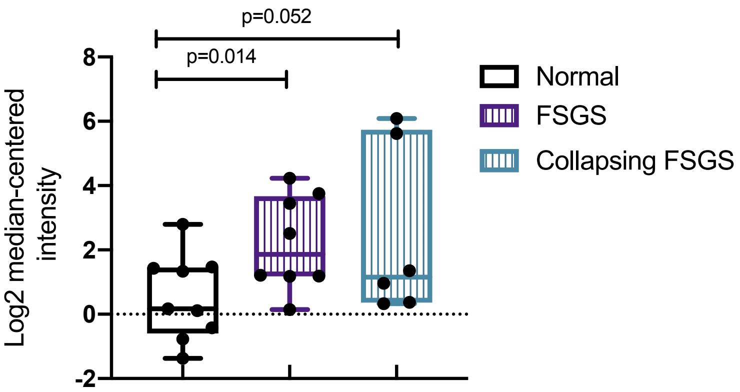

S. Fig. 4: Expression of SPP1 in human FSGS. Glomerular *SPP1* mRNA expression in kidneys of patients with primary classic FSGS (n=8), collapsing FSGS (n=6) and normal control (n=9) in Nephroseq database (*nephroseq.org*). Dataset from Hodgin et al.
